# Loss of microbial diversity from human–microbe coevolution: immune, epigenetic, and enteric nervous system mechanisms linking dysbiosis to neuropsychiatric and neurodegenerative diseases

**DOI:** 10.3389/fnins.2026.1952549

**Published:** 2026-10-02

**Authors:** Hajar Heidari, Michael Brimacombe, David A. Lawrence

**Affiliations:** 1 College of Integrated Health Sciences, University at Albany, State University of New York, Albany, NY, United States; 2 The Wadsworth Center, New York State Department of Health, Albany, NY, United States; 3 Department of Pediatrics, UConn School of Medicine, Farmington, CT, United States; 4 Connecticut Children's Research Institute, Hartford, CT, United States; 5 College of Integrated Health Sciences, University at Albany, Albany, NY, United States

**Keywords:** autoimmune diseases, chemokines, cytokines, dysbiosis, environmental pollutants, genetics, heritability, inflammation

## Abstract

*Homo sapiens* are the only species of humans still existing, as far as we know. *Sapiens* became the “sensible, shrewd, knowing” species of humanoids (the *Homo* genus) with assistance from the gut microbiota that evolved alongside and within *Homo sapiens,* providing metabolites and assisting the gut-brain axis to aid brain expansion and function. This review discusses coevolution, how dietary and environmental changes have influenced the importance of the gut microbiota, the microbiome-gut-brain axis (MGBA) and related epigenetic changes, and how current procedures and products may alter the beneficial commensal relationship and contribute to increased incidence of disorders such as asthma, autoimmune diseases, obesity, and neuropathology. Immune and endocrine homeostasis are tightly linked to the MGBA, and industrialization, by dispersing more chemicals into the environment, is upsetting the balance. Although brain laterization influences on immunity and neurological disorders are reported, there is no definitive linkage between brain hemisphere effects on gut microbiota or the MGBA. Brain laterality (left/right hemisphere differences) affects physical and mental stress sensitivity, but linkage of MGBA and metabolites to autoimmune neurological diseases remain speculative. Loss of microbiota diversity and altered proportions of certain bacterial species due to environmental stressors may lead to increased numbers of opportunistic and pathogenic bacteria (dysbiosis) creating metabolites having fewer beneficial and more detrimental effects across multiple host organs. Diet, environmental toxicants, and stress are presented as influences on the current increased incidence of some immune-associated disorders, autoimmune diseases, and neuropathy. Increased toxic insults are hypothesized to coincide with immune disorders based on gut microbiota changes. Within the CNS itself, complement- and immunoglobulin-mediated synaptic pruning has emerged as a convergent mechanism linking peripheral immune dysregulation to synaptic vulnerability in neurodegenerative and neuropsychiatric disease, suggesting that enteric nervous system and MGBA disruption may act upstream of these processes. Genetic and genomic aspects of the host-microbiome relationship are also discussed.

## 1 Introduction

The coevolution of *Homo sapiens* (the Genus species of current humans) and their associated microbial communities has shaped human physiology, immunity, and development over millions of years, resulting in a tightly integrated meta-organism or “holobiont” (Dethlefsen et al., 2007; Sarkar et al., 2024; Santoro et al., 2020; Ma et al., 2023). *Homo sapiens* survived even though other animals could run faster and had more efficient senses (smell, sight, and hearing). Once erect, the *Homo* species learned to use the forelimbs for other tasks, such as making and using tools for defense and hunting, which increased the amount of meat (protein) in their diet. The use of fire became a tool for keeping warm and cooking, helping to tenderize meat and reduce the presence of pathogens. The acquisition of skills in mastering fire, cooking, and cereal cultivation influenced both human and microbial evolution through their shared evolutionary pathway (Tannock, 2024). Cooking and diet evolutionarily fueled the growth of the human brain (Gibbons, 2007) and affected the length of the GI tract, the partitioning of bacterial species into sections, and the epithelial barrier’s absorption/transport functions, providing more nutrients for brain development (Milton, 2003).

The abilities of the *Homo* species are thought to be largely based on evolved improvements in cognitive capacity with increased brain size and function. Cognition remains the predominant biological trait that enables *Homo sapiens* to survive and thrive. Many other species were coevolving alongside the *Homo* species and adapting to their changes. Some resided within the evolving *Homo* species and accommodated dietary changes. The microbes in the gut coevolved and adapted, and as they were fed, they produced metabolites that helped the *Homo* species further evolve. Some foods would not be digestible without the numerous microbes in the gut (the gut microbiota). The enlarging brain with hominin evolution is suggested to require an obligatory glucose demand which was likely accompanied by a dietary change to more fruits and vegetables and co-evolution of the gut microbiome (Brand-Miller et al., 2026). The entire host, especially the brain, benefits from this commensal relationship between microbes and host cells (microbiomes). There is bidirectional communication between the gut microbiome and the brain through nerves and metabolites (the microbiome-gut-brain axis, MGBA). The MGBA delivers signals to the brain’s hypothalamus via hormones, nerves, and metabolites to control hunger and satiety (van de Wouw et al., 2017). The vagus nerve is the main bidirectional parasympathetic pathway between the brain, the heart, and the gastrointestinal (GI) system; it plays a major role in controlling mood, immune response, digestion, and heart rate (Breit et al., 2018).

The GI system also has the enteric nervous system, which is numerous neurons embedded in the lining of the GI system and is sometimes referred to as “brain in the gut”; the enteric nerves control digestion, help move digested food through the GI tract to the rectum and affect immune cells participating in the GI system (Sharkey and Mawe, 2023). The enteric nervous system and enteric glial cells affect the human GI barrier and the microbiota (Dowling et al., 2022). The enteric nervous system and the glial cells likely may also have been involved in the length of the GI system (Puzan et al., 2018).

The genes of the microbes in the gut far outnumber those of the host. Similar to the gut microbiome, there are microbiomes at other body sites (Dunn et al., 2020; Weyrich, 2020; Fellows Yates et al., 2021), such as the mouth, skin, lungs, and even the brain, that assist with host functions, including defense against pathogens. Within specialized microbiome areas, microbes are closely associated with host immune cells, and there are mutual beneficial interactions. However, if microbes escape from a microbiome due to physical damage to the barrier or exposure to toxicants, they can provoke systemic inflammation, as seen in sepsis (Chancharoenthana et al., 2023; Tian and Li, 2025).

Lifestyle, diet, contacts with xenobiotics (drugs and chemicals), medical practices, environmental exposures (air pollution and infections), stress, and climate change with more industrialization have been associated with a loss of ancestral microbial diversity and an increased incidence of endocrine and immune-associated chronic non-communicable diseases (NCDs) such as obesity, diabetes, allergies, autoimmune disorders (ADs), inflammatory bowel disease (IBD), multiple sclerosis, and even neuropsychiatric conditions (Aggarwal et al., 2022; Blaser and Dominguez-Bello, 2025; Reynoso-García et al., 2022; Mosca et al., 2016; Rook et al., 2017; Smith et al., 2022; Bello et al., 2018; Blaser, 2017; Blaser and Falkow, 2009).

Evidence from comparative studies across populations and primate relatives shows that humans seem to have lost many co-diversified gut symbionts, particularly in industrialized societies (Sanders et al., 2023; Rühlemann et al., 2024). Mechanistically, reduced microbial diversity impairs immune regulation, metabolic homeostasis, and resilience against pathogens (Bradshaw, 2023; Sarkar et al., 2024; Ruff et al., 2020). The growing body of research highlights the urgent need to understand and restore lost microbial diversity to address the modern epidemic of chronic diseases (Aggarwal et al., 2022; Mosca et al., 2016; Bello et al., 2018).

While most evidence supports the link between diversity loss and disease risk via disrupted coevolutionary relationships (Dethlefsen et al., 2007; Ma et al., 2024), some reports caution that causality is complex, and host-related genetic or environmental filtering may also play roles. Population-specific effects exist, and more mechanistic, environmental and epidemiologic work is needed (Belizário and Napolitano, 2015; Ma et al., 2024).

## 2 Microbes within humans

Phylogenetic evidence supports co-speciation of gut bacteria families (Bacteroidaceae and Bifidobacteriaceae) with *Homo* species over 15 million years and with bacterial and host divergence times occurring in concert (Moeller et al., 2016). This coevolution shaped fundamental physiological systems including immune system development (Lee and Mazmanian, 2010; Zhao and Elson, 2018; Kim et al., 2025), digestive adaptations (Dunn et al., 2020), spatial compartmentalization of the gut (Walter and Ley, 2011), and metabolic capabilities including starch digestion through both microbial acquisition of amylase-binding proteins (Fellows Yates et al., 2021) and host AMY1 gene copy number expansion (Walter and Ley, 2011).

Microbiotas promote major histocompatibility complex (MHC) class I-related protein 1 (MR1) expression on macrophages which enables maintenance and activity of mucosal-associated innate T (MAIT) cells providing immunity to regulate both the microbiota and infecting bacteria (Tastan et al., 2018), especially in the lungs (Deng et al., 2026). The *Firmicutes* and *Bacteroidetes* phyla have been found to dominate the gut microbiome in healthy subjects (Khalil et al., 2024). The *Firmicutes* and *Bacteroidetes* phyla were officially renamed Bacillota and Bacteroidota by the International Committee on Systematics of Prokaryotes; however, many studies still use the previous names. The proportions of bacterial phyla in healthy human guts are shown in Figure 1.

**Figure 1:**
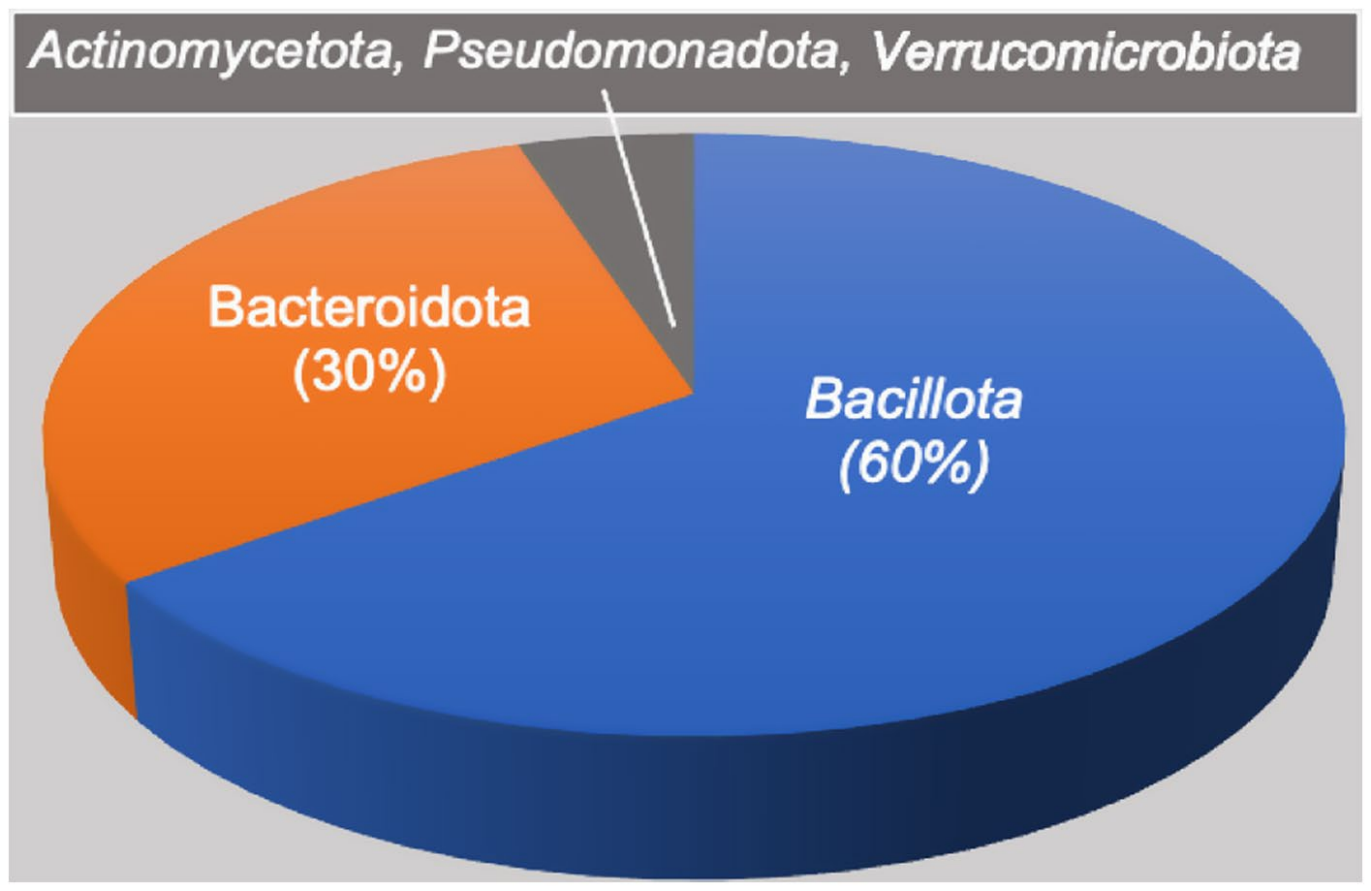
Proportions of the more abundant phyla in GI tract of healthy humans. Bacteroidota and Bacillota constitute about 90% of the composition. *Faecalibacterium prausnitzii* of the Bacillota phylum is generally considered the most abundant and prevalent bacterial species (Miquel et al., 2013) often representing over 5% of the total of about 400 species in the healthy adult human gut. The Bacillota phylum affects SCFA production (butyrate, acetate) and mucosal maintenance. The Bacteroidota phylum affects carbohydrate fermentation, mucin degradation, and low-grade LPS production. The Actinomycetota phylum affects immune development, acetate/lactate production and early-life development.

*Firmicutes, Bacteroidetes*, and *Actinobacteria* are the most abundant bacterial phyla in the gut (Syromyatnikov et al., 2022). The infant’s gut microbiota primarily originates from the maternal birth canal and breast milk. The *Firmicutes/Bacteroidetes* ratio is often used as a biomarker of gut dysbiosis, as observed in obesity (Magne et al., 2020); however, observed heterogeneity across studies precludes its clear use for diagnosis (Pinart et al., 2021). The activities of gut bacterial species in immune homeostasis and the GI barrier are listed in Table 1. *Bifidobacterium* is a widely distributed commensal bacterial genus that displays beneficial pro-homeostatic and anti-inflammatory immunomodulatory properties; depletion or absence of *Bifidobacterium* in humans and model organisms is associated with ADs and impaired immune homeostasis (Gavzy et al., 2023).

**Table 1:** Bacterial species in the human gut and their beneficial or detrimental influences.

Phylum	Bacterial genera species*	Mechanism/reference
Bacillota	*Faecalibacterium prausnitzii* *Roseburia, Ruminococcus, Lactobacillus, Clostridium, Lawsonibacter, Eubacterium, Butyricoccus, Subdoligranulum, Anaerobutyricum, Lactobacillus*, *Ruminococcus*, *Eubacterium*, *Christensenella minuta, Bifidobacterium, Dialister*	Many species affect butyrate production, the anti-inflammatory SCFA crucial for colonocyte health, gut lining integrity, and immune regulation. Some are pathobionts like *C.difficile, C.perfringens, Enterococcus, Peptostreptococcus, Streptococcus, Finegoldia* and *Blautia*
Bacteroidota	*Bacteroides, Prevotella, Parabacteroides, Alistipes, Ordoribacter, Barnesiella, Capnocytophaga, Tannerella*	Bacteroides are vital for complex carbohydrate digestion and immune development as essential commensals; however, like other commensals outside of gut they can trigger significant health issues (Zafar and Saier, 2021).
Actinomycetota	*Bifidobacterium, Collinsella, Eggerthella, Aldercreutzia, Slackia, Rothia, Actinomyces* *Gordonibacter*	*Bifidobacteria* ferment prebiotic fibres into acetate and lactate, support gut lining health and immune modulation, play a critical role in infant gut development (Stuivenberg et al., 2022).
Pseudomonadota	*Escherichia* (*E.coli*), *Klebsiella, Salmonella, Enterobacter, Helicobacter, Sutterella, Haemophilus, Shigella, Hafnia, Bilophila, Desulfovibrio, Morganella, Citrobacter*	*Pseudomonadota* genera species include many of the gut’s opportunistic and pathogenic bacteria, and they are associated with IBD, chronic gut dysbiosis, post-antibiotic states, and poor microbial diversity (Winter and Bäumler, 2023).
Verrucomicrobiota	*Akkermansia muciniphila*	*A. muciniphila* maintains intestinal barrier integrity, ameliorates metabolic and inflammatory disorders, and enhances anti-tumor immune responses (Ding et al., 2025).

*Partial list of commensals, opportunistic and pathogenic bacterial species in five phyla.

Viral infections can be related to selection pressure that induces MHC diversity and to endogenous retroviral insertions that affect gene expression (Jeffery and Bangham, 2000; Van Blerkom, 2003). The diversity of the immune system reflects the coevolution of symbiotic cooperation of commensal microbes and competitive pressures from genetic adaptations (Lee and Mazmanian, 2010).

After the Industrial Revolution, disruption to the coevolutionary relationships of the microbiome composition and the host, known as dysbiosis, seems to have become more common (Weyrich et al., 2015, Weyrich, 2020) and has been linked to higher incidence of ADs (Lee and Mazmanian, 2010; Mangalam et al., 2021; Mousa et al., 2022), metabolic diseases (e.g., obesity) (Walter and Ley, 2011; Yang and Kweon, 2016) and systemic conditions (Weyrich, 2020; Yoo et al., 2020), which supports the ongoing health significance of microbial-human coevolution.

Dysbiosis involves the loss of beneficial microbes, reduced diversity, or overgrowth of pathogens. This imbalance triggers chronic inflammation and immune dysfunction (Zhao et al., 2023). Early disruption of the microbiota by antibiotics or environmental stressors alters microbe-immune interactions, increasing the prevalence of ADs and allergies (Rasmussen et al., 2018; Kim et al., 2025). Specific microbes and microbial products have been suggested to hamper proper immune education in early life, leading later to a higher incidence of type 1 diabetes and other ADs and allergic diseases (Blaser, 2017; Sonnenburg and Sonnenburg, 2019; De Luca and Shoenfeld, 2019; Zimmer, 2025). Loss of gut microbial diversity in industrial populations is suspected to contribute to the development of chronic diseases, underscoring the importance of studying our ancestral gut microbiome (Wibowo et al., 2021).

Population-specific differences in disease susceptibility seem to reflect distinct microbial evolutionary histories influenced by past environmental exposures and genetic adaptations such as lactase persistence and AMY1 expansion (Walter and Ley, 2011; Weyrich, 2020). In the gut, extensive metabolic networking allowed bacteria to degrade and ferment components of food and human secretions (Tannock, 2024), with microbiomes contributing to host nutritional buffering (Amato et al., 2019).

Global differences in environmental toxicants and stresses may convert or allow commensals to become pathogens along with exposure to new environmental pathogens and other foreign antigens. Immunity to the new antigenic determinants have been increasing the human leukocyte antigen (HLA) haplotype differences in human populations (Arrieta-Bolaños et al., 2023). As HLA specificities increase so too may the ability to recognize epitopes of self-constituents that may influence the development of ADs (Liu et al., 2025; Harroud and Hafler, 2023), which include HLA alleles associated with autoimmune neurological diseases (Muñiz-Castrillo et al., 2020; Ghafouri-Fard et al., 2022). The study by Miclescu et al. (2024) found that carriers of A*02:01-B*15:01-C*03:04-DRB1*04:01-DQB1*03:02 haplotype have an odds ratio of 9.3 for developing clinically significant persistent neuropathic pain after nerve injury and surgery. Genome-wide association studies (GWAS) have shown that variants of HLA molecules are among predominant contributors to ADs (Matzaraki et al., 2017). GWAS identified a protective *IL-23R* variant in Crohn’s disease (Duerr et al., 2006), and subsequent studies implicated IL-23R in multiple ADs which lead to IL-23-targeted therapies (Krueger et al., 2024).

The adaptive arm of this coevolved relationship is anatomically distributed: tertiary lymphoid structures containing germinal centers (GCs) form directly at barrier organ sites hosting a local microbiome, including the gut-associated lymphoid tissue (Peyer’s patches and isolated lymphoid follicles), the upper airway, lung, and skin, providing a first layer of local, largely IgA-biased humoral immunity that maintains microbiome homeostasis before more extensively selected, higher-affinity IgG responses are mounted in draining lymph nodes (Foster and Nowosad, 2026). This tissue-specific, GC-mediated compartmentalization of host-microbe immune surveillance offers a structural counterpart to the coevolutionary pressures described above. As the gut microbiota diversified alongside the host, so too did dedicated local lymphoid architecture that allows tolerance of commensals while retaining rapid response capacity against pathogens. Loss or dysregulation of these barrier germinal centers is implicated in autoimmune conditions such as inflammatory bowel disease (Foster and Nowosad, 2026).

Beyond HLA-linked susceptibility, the coevolutionary relationship between host genome and microbiota is likely mediated by a broader layer of gene–environment interaction and epigenetic regulation. Host genetic variation shapes, which microbial taxa can colonize and persist with polymorphisms in *FUT2* (the “secretor” gene), alter intestinal mucosal glycan availability; these changes are strongly associated with the abundance of *Bifidobacterium* species (Wacklin et al., 2011), while variants in the nucleotide-binding oligomerization domain-containing protein 2 (NOD2) innate immune signaling pathway, including NOD2 itself and CARD9, are among the most consistently replicated genetic risk loci for Crohn’s disease (Ashton et al., 2023). Microbial metabolites may act on the host genome epigenetically; the short-chain fatty acid butyrate, produced by gut fermentation of dietary fiber, inhibits host histone deacetylases (HDACs), potentially altering chromatin accessibility and gene transcription in intestinal and immune cells, with implications extending from metabolic disease to neurological degenerative disorders (Berni Canani et al., 2012). This bidirectional genome-microbiome dialogue may imply that similar environmental exposures (diet, toxicant, or stressor) can produce markedly different disease outcomes depending on an individual’s genetic background, and that loss of ancestral microbial diversity may itself remove epigenetic inputs that host genomes evolved to depend on.

## 3 Host intestinal epithelial cell interactions with microbes and immune cells

Stem cells of the intestinal epithelium have differentiated into specialized cells to maintain the epithelial barrier, provide a mucus layer for commensals, and signal immune cells to detect non-commensal microbes (Figure 2). Goblet cells are specialized guardians lining the intestine. They play a critical role in gut defense and immune regulation, with continuous mucus secretion creating a physical barrier that protects against pathogens and their escape from the gut while harboring symbiotic gut bacteria that live within the mucus and defend against pathogens (Tonetti et al., 2024). Tufted cells are chemosensory epithelial cells that act as “sentinels” for the gut’s immune system; they send microvilli (“tufts”) into the gut lumen to detect opportunistic microbes and signal adjacent cells, including type-2 innate lymphoid cells (ILC2 cells) with interleukin (IL)-25, acetylcholine (ACh), eicosanoids, and cysteinyl leukotriene C4 (LTC4). IL-25 and LTC4 are pro-inflammatory stimulators of ILC2 cells (Hendel et al., 2022). Paneth cells are secretory epithelial cells located at the base of small intestinal crypts of Lieberkühn; they secret antimicrobial proteins (e.g., defensins and lysozyme) (Vaishnava et al., 2008). The intestinal epithelial cell subsets, along with microbes and immune cells, have been found to exert multiple influences on the physiology of the GI tract and on immune and endocrine activities. Commensals, immune cells, and epithelial cells interact within the mucosal layer, maintaining and enhancing the barrier and defenses against invading opportunistic pathogens.

**Figure 2:**
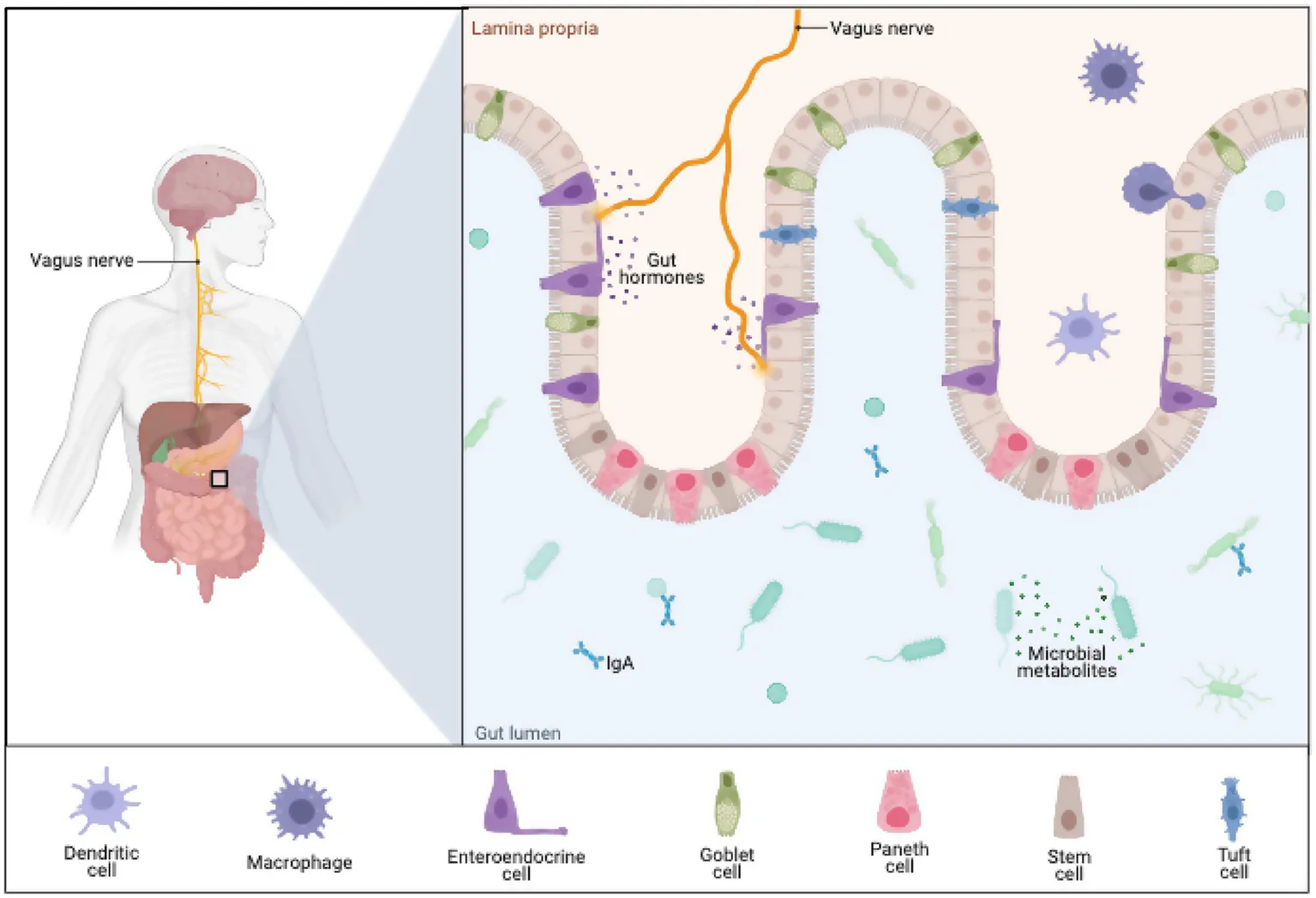
Microbiome-gut-brain axis (MGBA) and microbiota metabolites affect host through effects on endocrine, immune and nerve systems. Vagus nerve is main component of the parasympathetic nervous system enabling communication between the brain and visceral organs. Nerve-induced enteroendocrine cells are nutrient-sensing cells that release many gut hormones that regulate digestion, appetite, and metabolism, e.g., cholecystokinin, secretin, glucose-dependent insulinotropic polypeptide, glucagon-like peptide 1, peptide YY, ghrelin, and motilin (Gribble and Reimann, 2019). Paneth cells are secretory cells located in the crypts, adjacent to the intestinal stem cells, that produce antimicrobial peptides and proteins and other components that are important in host defense and immunity, e.g., alpha-defensins (HD5, HD6), lysozyme, and inflammatory mediators such as TNF-alpha and IL-17A. Goblet cells are specialized epithelial cells that function primarily to secrete mucus. Tuft cells are rare, specialized sensory epithelial cells found in the lining of the gastrointestinal and respiratory tracts, identified by a brush-like tuft of microvilli; they are “sentinels” detecting parasites, bacteria, and allergens, and initiate type 2 immune responses by secreting IL-25. Microbial production of metabolites effect immunity throughout the host. Gut hormones and microbial metabolites also effect brain function. Figure made with BioRender.com.

Environmental toxicants such as phthalates, Bisphenol A (BPA), heavy metals (lead, mercury), pesticides (glyphosate), and fine particulate matter (PM2.5) may often act synergistically with dietary stressors, heat, and psychological stress to disrupt the microbiome-gut-brain axis. These stressors reduce beneficial bacteria (e.g., *Lactobacillus*) and increase pro-inflammatory bacteria, promoting a “leaky gut” (Figure 3) and systemic inflammation by allowing microbes to escape, which may initiate systemic cell damage and autoimmune responses.

**Figure 3:**
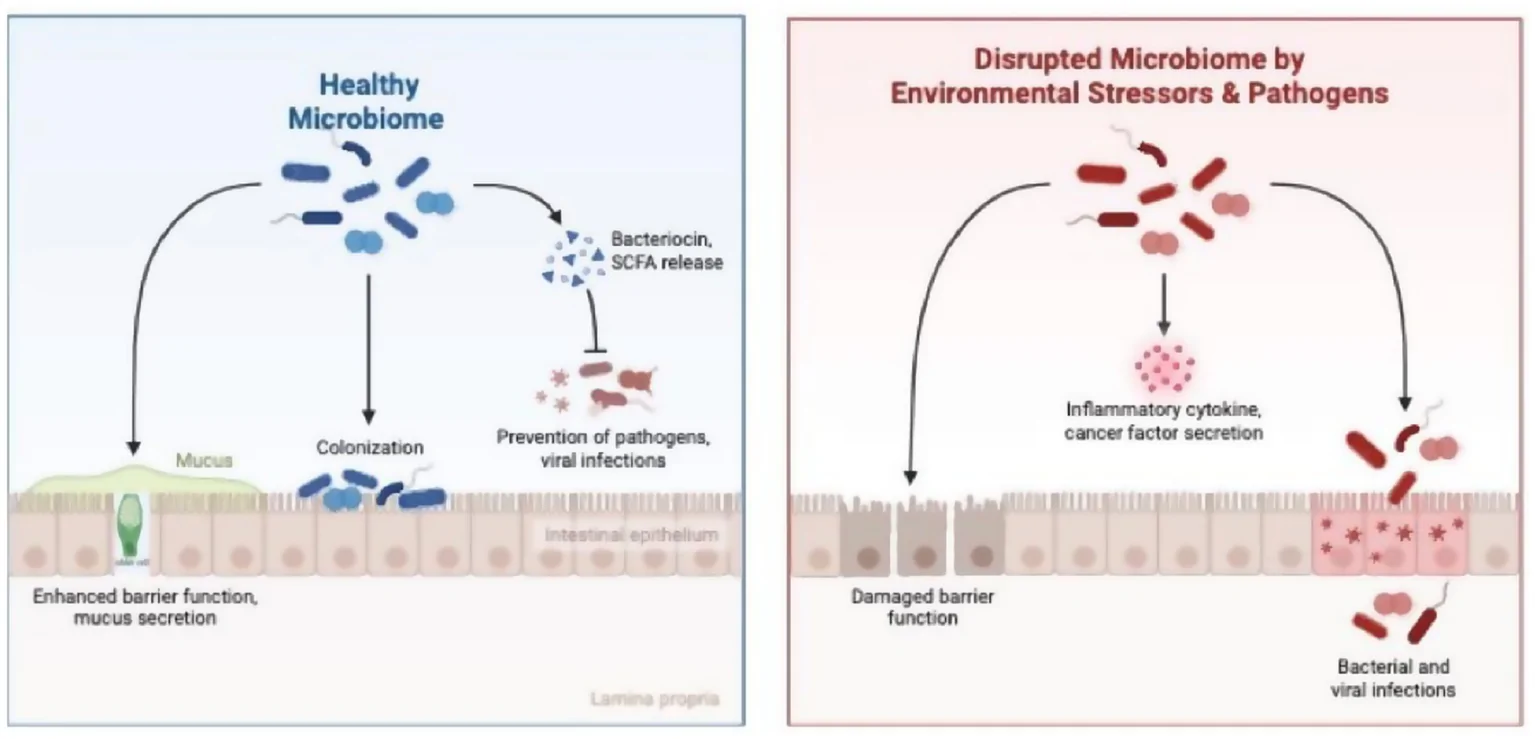
Influence of environmental stressors (e.g., microplastics and herbicides) and pathogens on gut microbiota. A healthy gut microbiome helps to maintain the intestinal endothelial barrier with mucin 2 (MUC2) as its skeleton component produced by goblet cells, which are induced by microbe-associated molecular (MAMPs); this creates the mucus barrier in which some microbes imbed. The healthy microbiome also helps defend against pathogens; the commensals produce bacteriocin (antimicrobial peptides to eliminate or inhibit closely related pathogens to protect their secure ecological niche. With a disrupted microbiome (dysbiosis), gut microbes may escape the gut and enter the blood to cause host inflammation and OS. Figure made with BioRender.com

## 4 MGBA and brain laterality

Direct evidence specifically linking brain hemisphere differences (lateralization) to differential effects on the gut microbiome remains limited. Most studies focus on the bidirectional communication between the entire central nervous system and the gut, rather than isolating the roles of the left versus right hemispheres. Although brain laterality effects on behaviors, including psychiatric disorders, and immunity have been reported (Mundorf et al., 2021; Cappa, 2025), there have been few studies researching laterality influences on connections with microbiomes, the MGBA, and immune responses that are associated with brain disorders. However, there are indirect connections, such as differential brain hemisphere differences in stress, which usually involve the right hemisphere to a greater extent (Ocklenburg et al., 2016), and immune responses, with the left hemisphere associated with enhancing or patenting immune activity (Meador et al., 2004). Brain laterality differences are indicated in Table 2. Note that interactions among microbial metabolites, immune pathways, endocrine signaling, and neural pathways may affect these results.

**Table 2:** Brain hemisphere influences.*

Feature	Left hemisphere association	Right hemisphere association
Autonomic focus	Predominantly Parasympathetic (rest/digest)	Predominantly Sympathetic (fight/flight)
Gut impact	Promotes regular motility and mucus secretion	May slow motility or increase permeability via cortisol/NE
Neurochemical expression	Usually associated with higher dopamine receptor activity (Fitzgerald, 2012; Fuertinger et al., 2018)	Usually associated with more dominant norepinephrine systems (Oke et al., 1978; Kruglikov et al., 1992).
Microbiome link	Associated with specific gray matter volume in the frontal cortex (in schizophrenia) Since the autonomic nervous system (ANS) regulates gut motility, intestinal transit, and secretion, hemispheric imbalances (such as those following a stroke) can lead to asymmetrical “top-down” signaling that shifts the gut environment and microbial composition.	Associated with diversity markers in the postcentral gyrus
Chronic stress		Right prefrontal cortex more significantly affected. Because stress directly alters the gut microbiome, hemispheric differences in how an individual processes stress may indirectly lead to different “microbial signatures” in the gut. (Ocklenburg et al., 2016)
Immunity	Generally associated with stronger immunity and enhanced immune reactivity (Sumner et al., 2011).	Often has a more immunosuppressive or regulatory influences. (Stoyanov et al., 2012)

* Brain hemisphere differences define brain laterality. Most humans are right-handed, which is primarily due to the left hemisphere. Although a substantial number of patients with ADs have been reported to be left-handed (Gardener et al., 2009; Searleman and Fugagli, 1987; Morfit and Weekes, 2001), the % has been well substantiated. Handedness is partially genetic, but it is not determined by a single gene. It is a polygenic trait influenced by at least 40 different genes, with studies estimating a genetic heritability of around 25%. Environmental factors, such as prenatal development and cultural influences, also play a significant role. (McManus et al., 2013).

Communications between the brain and gut microbiota are bidirectional, with brain laterality influencing them (Sumner et al., 2011; Dantzer, 2018; Leunig et al., 2025; Meador et al., 2004; Neveu, 2002). Since both stress and immune responses affect the gut microbiota, it seems likely that brain laterality affects the gut microbiota; however, research on laterality and gut microbiota needs more correlational and causal data to determine whether brain laterality significantly affects health through changes in the gut microbiota. Laterality differences in neurochemical expression were observed in female and male mice after exposure to lead (Pb) ± prenatal stress (PS) (Cory-slechta et al., 2013).

## 5 Human–microbe coevolution: foundations and loss of microbial diversity

Multiple studies provide evidence that humans have co-evolved with complex microbial communities across body sites (gut, skin, oral cavity) over millions of years (Dethlefsen et al., 2007; Sarkar et al., 2024; Ma et al., 2024; Davenport et al., 2017; Santoro et al., 2020; Ma et al., 2023). Key gut lineages such as Bacteroidaceae and Bifidobacteriaceae show codiversification with hominids (Ma et al., 2024; Sanders et al., 2023; Suzuki et al., 2021), while host-specific adaptations are evident in both microbes and humans (Rühlemann et al., 2024). The holobiont concept frames humans as meta-organisms whose health depends on these ancient partnerships (Reynoso-García et al., 2022; Sarkar et al., 2024).

Industrialization seems to have driven a dramatic reduction in microbiome diversity (Aggarwal et al., 2022; Reynoso-García et al., 2022; Mosca et al., 2016; Smith et al., 2022; Santoro et al., 2020; Bello et al., 2018). Comparative studies suggest that westernized populations have lost many ancestral taxa found in rural or traditional societies—and even more so than in non-human primates (Thingholm et al., 2021; Sanders et al., 2023; Gupta et al., 2017; Rühlemann et al., 2024). Humans in industrialized societies harbor the lowest gut bacterial diversity of any primate studied. Moeller (2017) suggests that this depauperating began deep in human evolutionary history but has accelerated dramatically with modern lifestyles and coincides with the increased incidence of immune-associated disorders (Miller, 2022).

The most striking recent evidence comes from Carter et al. (2023), who performed ultra-deep metagenomic sequencing of Hadza hunter-gatherers in Tanzania and identified 124 gut-resident species that are diminishing in industrialized populations, with 44% of recovered genomes absent from existing reference datasets. A multi-population comparison by Rosas-Plaza et al. (2022) across hunter-gatherer, agricultural, agropastoral, pastoral, and urban groups suggests that diversity loss tracks a gradient of industrialization, with bacterial composition reflecting the transition from traditional to modern lifestyles.

Current possible drivers affecting loss of microbiota diversity include: diet, antibiotics, excessive sanitation, cesarean delivery, formula feeding, and increased exposure to environmental stressors. These changes in early life are particularly consequential (Vangay et al., 2015) and may have additive, synergistic, and/or antagonistic effects (Figure 4). These associations have been framed as an “invisible extinction” characterized by the depletion of our commensals and the erosion of metabolism, immunity, and cognition (Joos et al., 2025). The microbiota changes have also been suggested to relate to the hygiene hypothesis as “Old Friends” depletion (Rook et al., 2017), leading to fewer organisms that induce immunoregulatory circuits, and their absence undermines tolerance mechanisms, contributing to ADs. It has been suggested that diversity loss reduces the stability and adaptability of the gut ecosystem and reverses evolutionary complexity (Mosca et al., 2016).

**Figure 4:**
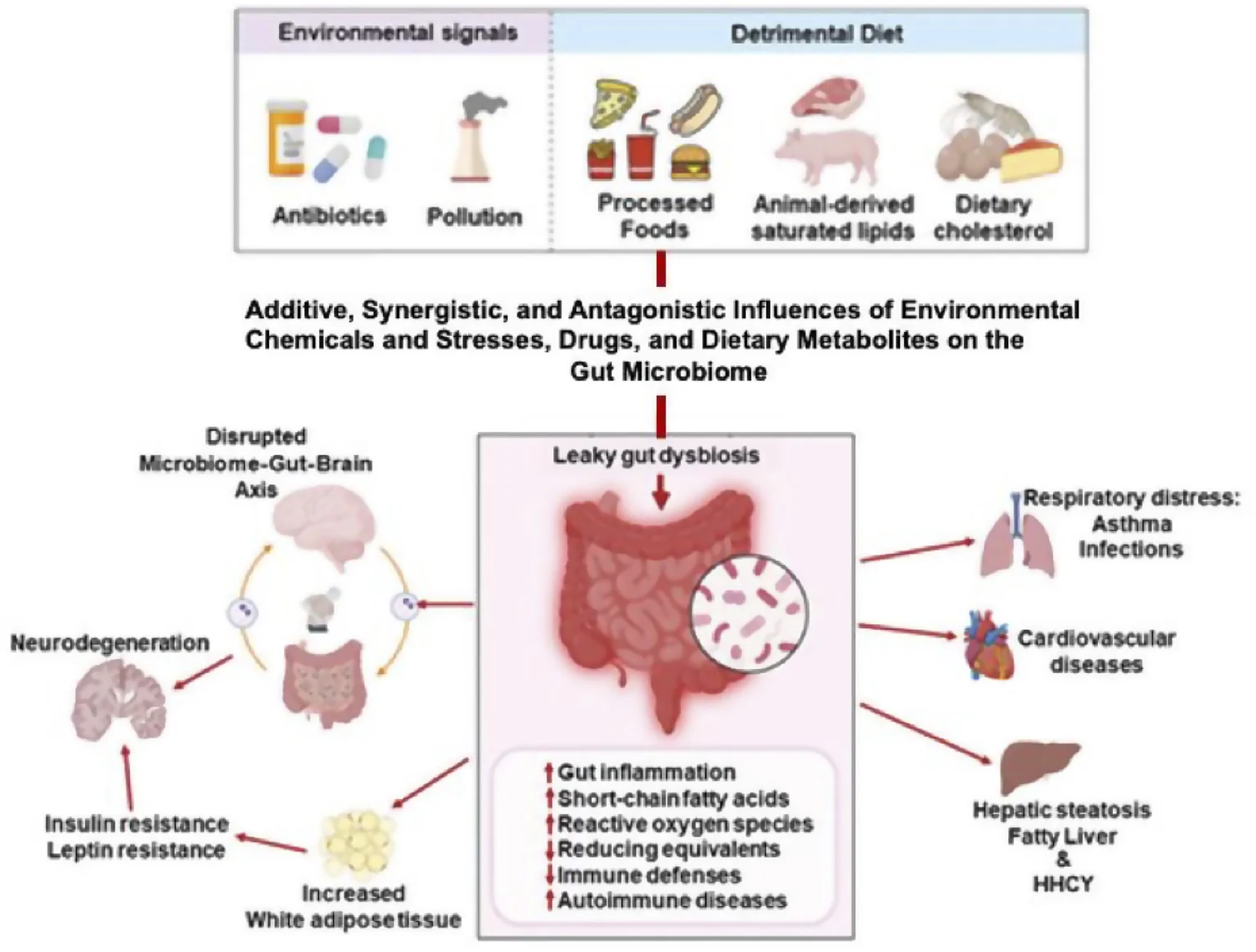
Global differences in environments, diets and lifestyles differentially affect the gut microbiome. Gut dysbiosis leads to loss of gut mucus and epithelial barrier. Leakage of microbes into blood elevates inflammation and OS which alters the function of many organs. Dysbiosis may lead to increased prevalence of infections, cancer, autoimmune diseases, and/or neuropsychiatric and neurodegenerative diseases. Figure made with BioRender.com

Real-world exposure scenarios typically involve chronic exposure, low-dosage, and mixtures of pollutants (e.g., PM2.5, BPA, Cd, and microplastics), yet most experimental designs focus on single high-dose pollutants. These simplified designs and related models overlook the synergistic or antagonistic interactions among pollutants, as well as possible interactions with host factors such as age, sex, diet, and genetics that shape microbiota resilience (Ma et al., 2026). Notably, most existing studies focus on single pollutants, whereas real-world exposures often involve mixtures. Co-exposure to agents such as heavy metals and persistent organic pollutants (POPs) can have additive or even synergistic effects on the gut microbiota, leading to more severe dysbiosis and barrier disruption. Furthermore, host factors such as diet, genetics, age, and sex influence susceptibility to microbial alterations.

For instance, high-fiber diets may mitigate pollutant-induced depletion of short-chain fatty acids (SCFAs), while aging is linked to reduced microbial resilience. SCFA deficiency, primarily butyrate, acetate, and propionate, results from reduced dietary fiber intake or microbial imbalance (dysbiosis). Dysbiosis has been found to weaken the gut barrier, trigger inflammation, and contribute to metabolic syndromes, inflammatory bowel disease (IBD), and cognitive decline (Lange et al., 2023; Wang et al., 2023). Genetic variations in immune or detoxification pathways may also shape individual responses. Future studies should consider adopting multi-pollutant models and account for inter-individual variability to better reflect real-world conditions. Advanced computational modeling and exposome–microbiome correlation networks may help capture the complexity of environmental exposure biology.

## 6 Disease associations with reduced microbial diversity

Low microbial diversity (“dysbiosis”) is often associated with a wide range of diseases and environmental pollutants affecting multiple systemic areas and processes. Gut microbiota associated with certain pathologies are reported in Table 3.

**Table 3:** Gut bacterial species associated with autoimmune diseases and other pathologies.

Disorder	Bacterial genera species*	Mechanism/reference
Systemic lupus erythematosus (SLE)	*Enterococcus gallinarum* *Ruminococcus gnavus* *Clostridioides diffcile, staphylococcus aureus, Streptococcus, Bacillus anthracis* *Streptococcus* *Lactobacillus salivarius*	When 𝐸. 𝑔𝑎𝑙𝑙𝑖𝑛𝑎𝑟𝑢𝑚 translocate, inflammatory Th17 cells and produces autoimmune-related antibodies, such as anti-dsDNA and anti-Sm are produced (Bagavant et al., 2021). *R. gnavus* was elevated 5-fold in the gut microbiota of 61 patients with SLE compared to that in healthy control and was strongly associated with SLE disease activity including anti-dsDNA (Azzouz et al., 2019, 2023).
Rheumatoid arthritis (RA)	*Prevotella copri, Subdoligranulum didolesgii, Eggerthella lenta Clostridium asparagiforme*, *Gordonibacter pamelaeae*, *Eggerthella lenta*, and *Lachnospiraceae bacterium*	Expansion of intestinal *Prevotella copri* correlates with enhanced susceptibility to arthritis (Scher et al., 2013). *Subdoligranulum*. Promotes IgA and IgG autoantibodies (Chriswell et al., 2022) Antibodies against citrullinated protein antigens (ACPAs) (Holers et al., 2018). High levels in RA were normalized with treatment (Zhang et al., 2015).
Multiple sclerosis (MS)	*Akkermansia muciniphila, Methanobrevibacter, Blautia, Ruminococcus* *Faecalibacterium, Prevotella, Butyricicoccus* Lachnospiraceae, *Bifidobacterium*, *Roseburia*, *Coprococcus*, *Butyricicoccus*, *Lachnospira*, *Dorea*, *Faecalibacterium*, *Prevotella, Monoglobus pectinyliticus*	Increased levels of pro-inflammatory bacteria *Akkermansia muciniphila, Methanobrevibacter,* and *Blautia* and a decrease in protective, anti-inflammatory species *Faecalibacterium, Prevotella,* and *Butyricicoccus* (Ordoñez-Rodriguez et al., 2023) Increased relative abundance and prevalence of *Monoglobus pectinyliticus* in MS; normal IgA-coating patterns microbiota were disrupted at the onset of MS, as well as those (particularly *Akkermansia muciniphila*) whose IgA-coating became more aligned with controls after therapy (Gupta et al., 2025).
Inflammatory bowel disease (IBD)	*Runinococcus gnavas, Ruminococcus torques, Roseburia hominis, Faecalibacterium prausnitzii*	Therapeutic options for manipulating the altered gut microbiota, such as probiotics and fecal microbiota transplantation have been considered (Nishida et al., 2018). Decrease in butyrate-producing species *F. prausnitzii* and *R. hominis* (Fujimoto et al., 2013; Machiels et al., 2014)
Type 1 diabetes (T1D)	*Bacteroides dorei* and *vulgatus*, *Citrobacter rodentium*	Gut microbiome dysbiosis in type 1 diabetes (Zheng et al., 2018)
Cardiovascular disease	*Chryseomonas* sp., *Veillonella* sp., *Streptococcus* sp., *Porphyromonas gingivalis*, *Aggregatibacter actinomycetemcomitans*	The heart-gut axis is a new target for cardiovascular diseases, in that altering the gut microbiome is being explored therapeutically (Forkosh and Ilan, 2019) There is compelling evidence that metabolic syndrome relates to cardiovascular risk factors (Shariff et al., 2024) Through interdisciplinary research is needed for a comprehensive understanding of the gut-brain-cardiovascular axis (Singh et al., 2024)
Cancer	*Akkermansia muciniphila*	Modulates efficacy of PD-1-based immunotherapy against tumors (Routy et al., 2018)

The listed genera and species are likely only a partial list of those involved.

### 6.1 Disorders at body sites connected with microbes include

*Brain:* Dysbiosis triggers chronic systemic inflammation, alters neurotransmitter production (e.g., serotonin and GABA), and activates the vagus nerve, which is strongly linked to neurological and psychiatric disorders (Ataei et al., 2026). Complementing this gut-derived route, recent work shows that neuronal hyperactivity itself is sufficient to trigger complement component 1q (C1q) deposition and subsequent synapse loss in the adult hippocampus, and that this process recruits antibody-secreting B lymphocyte lineage cells and locally produced immunoglobulin M (IgM) that facilitate C1q tagging of vulnerable synapses; dampening neuronal hyperactivity in an Alzheimer’s disease mouse model reduced amyloid-beta and C1q accumulation and partially restored synaptic density (Crowley et al., 2026). These findings identify a humoral, B-cell-dependent arm of neuroimmune-mediated synaptic remodeling within the central nervous system itself, suggesting that immune cells and antibodies—whether recruited peripherally in the context of gut dysbiosis or generated locally at meningeal and hippocampal niches—converge on complement-mediated synapse elimination as a shared mechanism linking systemic immune dysregulation to neurodegenerative and neuropsychiatric vulnerability.

*Gut*: Obesity (high Firmicutes/Bacteroidetes ratio), IBD (low Firmicutes), diabetes/metabolic syndrome (Aggarwal et al., 2022; Gildner, 2020; Reynoso-García et al., 2022; Wilde et al., 2024).

*Skin*: Atopic dermatitis (loss of Actinobacteria) (Aggarwal et al., 2022).

*Lung*: Idiopathic pulmonary fibrosis progression (Aggarwal et al., 2022).

### 6.2 Microbe effects on physiological or pathological processes include

*Immune/inflammatory*: Allergies/autoimmunity rise linked to loss of “old friends” microbes (Bradshaw, 2023; Rook et al., 2017).

*Neuropsychiatric*: Depression/schizophrenia/anxiety share patterns of reduced butyrate producers (Thingholm et al., 2021).

*Inflammaging/immunosenescence*: Ties aging to shrinking core microbiome (Sarkar et al., 2024); mechanistically, loss of key mutualists/predators destabilizes ecosystems and impairs immune/metabolic regulation (Bradshaw, 2023; Mosca et al., 2016).

*Environmental toxicant-associated ADs*: Environmental toxicants such as phthalates, BPA, heavy metals (lead, mercury), pesticides (glyphosate), and PM2.5 have been found to act synergistically with dietary stressors, heat, and psychological stress to disrupt the microbiome-gut-brain axis. These stressors reduce beneficial bacteria (e.g., *Lactobacillus*) and increase pro-inflammatory bacteria, promoting a “leaky gut,” systemic inflammation, and neurotoxicity (Yu et al., 2025). The environmental factors account for up to 70% of all ADs (Vojdani et al., 2014; Zhao et al., 2019). Strong evidence links environmental agents, including solvents, crystalline silica, mercury, pesticides, and cigarette smoking, with the development of various ADs (Khan and Wang, 2018). However, significant knowledge gaps remain regarding potential cellular, molecular, and immunological mechanisms by which environmental agents contribute to the disease pathogenesis.

In addition to physical and environmental agents that can trigger and perpetuate an autoimmune response, the gut microbiome can also play a critical role in such responses. Dysbiosis of the gut, oral, and skin microbiomes has been linked to autoinflammation and tissue damage in susceptible individuals (Dehner et al., 2019). Thus, changes in the human microbiome could be a significant contributory factor in autoimmunity, as altered microbial composition can induce inflammation and loss of immune tolerance (De Luca and Shoenfeld, 2019). The composition and stability of the gut microbiome not only support nutrient absorption but also regulate the mucosal immune system; therefore, dysbiosis can result in multiple ADs (Li et al., 2018).

### 6.3 Key additive/synergistic stressors and toxicants affecting microbiota include

- Plastic-Associated Compounds (BPA, Phthalates, Microplastics): These compounds disrupt intestinal barrier integrity, reduce beneficial bacteria, and increase pro-inflammatory taxa, leading to metabolic and behavioral disorders.
- Heavy Metals (Lead, Cadmium, Mercury): These induce significant oxidative stress and reduce the abundance of SCFA-producing bacteria, leading to neurotoxicity and systemic inflammation.
- Pesticides and Herbicides (Glyphosate, Chlorpyrifos): These agents alter microbial diversity, inhibit beneficial bacteria, and contribute to neurodevelopmental toxicities.
- Airborne Pollutants (PM2.5, Nitrogen Oxides): Inhaled pollutants are associated with systemic immune responses and oxidative stress, acting alongside gastrointestinal contaminants to increase inflammation.
- Persistent Organic Pollutants (POPs): Examples like PCBs and PBDEs have been found to disrupt bile acid metabolism, affecting the gut microbiota’s role in immune regulation. These factors frequently act together; for example, high-fat diets can increase the absorption of environmental toxins, while stress-induced gut changes (altered motility) worsen the impact of pesticide exposure. This combined exposure often leads to immune-associated disorders, including inflammatory bowel disease, autoimmunity, and neurodegenerative diseases.

## 7 Microbes and autoimmune diseases

Autoimmune diseases have been increasing in the human population (Bradshaw, 2023; Rook et al., 2017). As noted above, the presence of increasing numbers and amounts of environmental toxicants may impact the onset of autoimmune diseases. Environmental pollutants have also been found to affect the microbiome and related families of microbes. Some research has been conducted to identify correlations and associations between the onset of specific autoimmune diseases and body site, microbiome type and specific microbes.

Autoimmune disorders such as IBD, RA, MS, PAH, SLE, T1D have been examined in relation to gut microbiomes and the presence of microbial families such as Firmicutes, Bacteroidetes and Actinbacteria. There have also been attempts to examine the loss of potentially beneficial microbes (dysbiosis). Note that in some studies, data on previous or existing exposures to environmental toxicants, known to impact the gut microbiome and its diversity, may not be accounted for and such latent associations may impact results. See Table 3 and the references listed therein.

## 8 Heritability of the microbiome

While most evidence supports a link between microbiome diversity loss and human disease risk via disrupted coevolutionary relationships (Dethlefsen et al., 2007; Ma et al., 2024), some reports caution that causality is complex, with both host genetics and environmental filtering playing roles (Ferretti et al., 2026). Note that not all changes are negative and population-specific effects exist with more mechanistic work needed (Belizário and Napolitano, 2015; Ma et al., 2024).

Studies that seek to examine the diversity loss and disease risk association in the human population must control for underlying host genetics, environment and diet, as well as define the set of microbes and related microbiome assumed relevant to measures of diversity loss. This is a large amount of data and requires careful, detailed study. If co-evolution is the focus, a longitudinal aspect to the studies may be required. Related statistical models often involve nested structures and the incorporation of latent variables. Complex models such as factor analysis, structural equation models and related gene-environmental models (Herrera-Luis et al., 2024) may be needed to identify significant effects. In most cases, host genetics have been the focus.

There is some evidence that the taxa and components that make up the microbiome are themselves at least partially heritable. This is in addition to modifications and co-evolution of the microbiome in response to host genetic mutation and heritability. More recently, advances in gene sequencing approaches and related computational tools have improved and expanded the ways in which the heritability of the microbiome can be measured and assessed and microbiome genome-wide association studies (GWAS) have been conducted (Dekkers et al., 2026). Twin studies in particular have allowed researchers to estimate microbiome-related heritability by controlling for host genetic and local environmental similarities (Goodrich et al., 2014). Indeed, some studies have also identified host genetic variation associated with microbiome composition and other microbiome quantitative traits, such as community-level microbial diversity measures, specific taxa levels, single-nucleotide polymorphisms (SNPs) and other genetic variants (Blekhman et al., 2015; Kolde et al., 2018).

The elements constituting the microbiome of an individual is influenced by many environmental factors, for example diet and medication use (Vich Vila et al., 2020). In regard to genetics, early family-based studies showed that individuals within the same family have more similar microbiomes than unrelated individuals, and that similarities in the gut microbiome are significantly higher in monozygotic twins than in dizygotic twins (Turnbaugh et al., 2009). The idea that the microbiome is at least partially heritable is also supported by quantitative trait locus mapping studies in mice (Benson et al., 2010).

## 9 Human host genetics and the microbiome

The human microbiome is a vast inter-related community of bacteria, viruses, archaea and fungi inhabiting the human body, especially the gut. The complex micro-ecosystem aids in digestion, and helps regulate the immune system, and, as noted above, alterations in the microbiome have been associated with obesity, autoimmune disorders and cancers (De Luca and Shoenfeld, 2019. It is challenging to measure such a complex system and it is often necessary to focus on specific elements to formally assess significant effects and correlations. In addition, potential confounders, including diet, medication use, age, genetics, lifestyle, geography, and socioeconomic factors require careful consideration.

### 9.1 Defining Microbiome summary traits that can be measured and correlated with host genetics

Due to the wide diversity present in the microbiome, there is often a need to define useful microbial elements that can be accurately measured and associated with the presence of specific host genes and genetic structures. This is an ongoing area of research and researchers have employed various approaches to defining microbiome traits appropriate for heritability analysis.

A basic approach is to assess the abundance of each individual taxa that may be interpreted as being associated with specific traits, though many of these individual microbial measures are correlated (Gloor et al., 2017), often due to the heritability of structural variants in the microbial genome (Zhernakova et al., 2024).

To better reflect this complexity, one common approach involves using microbial community summaries derived from statistical dimension reduction techniques such as principal coordinate analysis to identify major axes of variation within microbial communities. The resulting components, which are weighted averages of the original set of taxa expressions, can be used as quantitative traits of interest in heritability studies (Goodrich et al., 2014).

Diversity measures, such as the Shannon diversity (Lozupone et al., 2012) measure or the alpha diversity measure the complexity of a microbial community by essentially assessing the relative abundance of different taxa present, often within a specific region of the gut or other body area.

### 9.2 Statistical methods and study designs

There is often a need to control for various aspects of the many factors affecting the host genes-microbiome relationship. One example is the use of twin studies. In this study design setting, host genetics are controlled for by the presence of identical twins, if the twins have been separated or subject to very different diets, the effects of environmental changes on the microbiome can be measured. Again, there is a need to specify the particular microbiome taxa that are of interest. Modelling the correlations between microbiome elements and genetic expressions and incorporating secondary mediating variables often requires complex structural equation models, as well as detailed factor analysis (Leong et al., 2020). Canonical correlation methods may also be useful (Deng et al., 2024). Canonical correlation essentially examines the correlation structure between host genetic principal components and microbiome principal components, especially useful in examining potential co-evolutionary patterns. To formally examine quantitative co-evolution, examining these structures longitudinally, often at specific time points, is required.

Given the large number of both host genes and microbial taxa involved, there is often a need to simplify the analysis, focusing on a chosen subset of variables which dominate the levels of variation observed. Statistical methods such principal components analysis create such summary variables that are weighted averages of the original variables (Johnson and Wichern, 2007). In attempting to model complex relationships among several sets of variables, other nested structures may be used including hierarchical methods, structural equations methods and canonical correlation.

Note that changes in host genetics occur fairly slowly, across the generations. The modeling of such changes may assume specific types of randomness in terms of how genes are passed on to the next generation. The simplest assumption is Mendelian randomization, which assumes a purely random mixing of host genes across the generations. This pattern may affect the heritability of the microbiome itself and the process of coevolution. Note that this may not be realistic if some genes are dominant or recessive or acquire mutations. If these assumptions do hold, randomization-based approaches can be used to develop relevant statistical tests. For example, Mendelian randomization is a statistical approach to testing for hypothesized associations between exposure and response by testing whether genetic variants associated with the exposure also correlate with a chosen response (Sanderson et al., 2022). Note that these results may be affected if additional variables are considered in the statistical modeling process.

Future studies should look to adopt multi-pollutant models and account for inter-individual variability to better reflect real-world conditions. Advanced computational modeling and exposome–microbiome correlation networks may help capture the complexity of environmental exposure biology.

### 9.3 Host gene regulation (RNA) designs

Studies have examined the relationship between host gene expression and the microbiome. Host transcription–microbiome associations have been studied using bulk RNA sequencing (RNA-seq), single-cell RNA sequencing (scRNA-seq) and spatial transcriptomics, to profile host gene expression, jointly analyzing these data with chosen microbiome features (both individual taxa and summary measures) to detect host–microbiota interactions (Lloyd-Price et al., 2019). Associations between microbiome composition and host gene expression have found host genes whose expression correlates with the levels of abundance of specific micro-bacterial taxa.

### 9.4 Host-microbiome co-evolution related designs

As the various microbiomes in the human body are sensitive to their local environments, including changes in nutrition, exercise or the onset of infectious disease, these changes may create selection pressures related to the composition of the microbiome. This may then affect how the microbiome interacts with the host and host related gene expression. Carefully structured study designs are required to model and interpret the many sources of variation The reviewed literature robustly supports the hypothesis that the long-term coevolution between *Homo sapiens* and their microbiota has been disrupted by modern lifestyles—leading to a measurable loss in microbial diversity that correlates with increased risk for a spectrum of chronic diseases (Aggarwal et al., 2022; Blaser and Dominguez-Bello, 2025; Dethlefsen et al., 2007; Reynoso-García et al., 2022). This disruption affects not only gut health but also immune function (the “hygiene hypothesis” and “old friends”), metabolism (obesity/diabetes), mental health (depression/anxiety), skin/lung conditions (atopic dermatitis/fibrosis), aging (“inflammaging”), and possibly even cancer risk (Thingholm et al., 2021; Sarkar et al., 2024; Mosca et al., 2016).

The strength of evidence is highest for gut-related metabolic/inflammatory diseases but extends across multiple organ systems due to the systemic influence of the microbiome on immunity to disease, body metabolism, and neurodevelopment (Aggarwal et al., 2022; Bradshaw, 2023). Causality can be difficult to prove definitively due to confounding factors such as age, genetics, environmental exposures, and diet; population based statistical models are required to provide formal modeling of the different levels of variation in microbial taxa levels correlated with host gene expression and genetic structures.

Note that such studies require replication and appropriate study designs. Many of the initial results regarding the microbiome, especially if not replicated and supported by population genetic insight, may be viewed as pilot studies, requiring future investigation.

## 10 Discussion

The reviewed literature robustly supports the hypothesis that the long-term coevolution between *Homo sapiens* and their microbiota has been altered by changes in lifestyles, which have led to a loss in microbial diversity associated with increased risks for a spectrum of chronic diseases. The strength of evidence is highest for gut-related metabolic/inflammatory diseases but extends across multiple organ systems. While causality is challenging due to potentially confounding factors (diet, medication use, age, genetics, lifestyle, geography, and socioeconomic factors), animal models and population comparisons provide convergent support; recent work has begun to support these claims.

The genetic history of the various microbiomes may be as long as the history of the species within which they exist. As environmental changes and access to nutrition have altered, so has the microbiome. The current focus, from a coevolutionary perspective, focuses on changes as humans have moved from an agricultural setting to an industrial society, with the associated changes in diet and nutrition and exposures to environmental pollutants. As noted above, these have been associated with an increase in the onset of various types of diseases and a reduction in overall microbial diversity, reflecting co-evolutionary selection pressures. Consensus points to an urgent need for restoration strategies targeting lost microbial functions as part of public health interventions.

## 11 Conclusion

The disruption and modification of ancient human–microbe partnerships through loss of microbial diversity, driven by industrialization, is strongly associated with rising rates of chronic inflammatory/metabolic/immune/psychiatric diseases worldwide. Restoration efforts to better target lost microbiome-based functions and taxa represent a potential emerging frontier for public health.
